# Inhibitors of Alphavirus Entry and Replication Identified with a Stable Chikungunya Replicon Cell Line and Virus-Based Assays

**DOI:** 10.1371/journal.pone.0028923

**Published:** 2011-12-19

**Authors:** Leena Pohjala, Age Utt, Margus Varjak, Aleksei Lulla, Andres Merits, Tero Ahola, Päivi Tammela

**Affiliations:** 1 Institute of Biotechnology, University of Helsinki, Helsinki, Finland; 2 Centre for Drug Research, Faculty of Pharmacy, University of Helsinki, Helsinki, Finland; 3 Division of Pharmaceutical Biology, Faculty of Pharmacy, University of Helsinki, Helsinki, Finland; 4 Institute of Technology, University of Tartu, Tartu, Estonia; Agency for Science, Technology and Research - Singapore Immunology Network, Singapore

## Abstract

Chikungunya virus (CHIKV), an alphavirus, has recently caused epidemic outbreaks and is therefore considered a re-emerging pathogen for which no effective treatment is available. In this study, a CHIKV replicon containing the virus replicase proteins together with puromycin acetyltransferase, *EGFP* and *Renilla* luciferase marker genes was constructed. The replicon was transfected into BHK cells to yield a stable cell line. A non-cytopathic phenotype was achieved by a Pro718 to Gly substitution and a five amino acid insertion within non-structural protein 2 (nsP2), obtained through selection for stable growth. Characterization of the replicon cell line by Northern blotting analysis revealed reduced levels of viral RNA synthesis. The CHIKV replicon cell line was validated for antiviral screening in 96-well format and used for a focused screen of 356 compounds (natural compounds and clinically approved drugs). The 5,7-dihydroxyflavones apigenin, chrysin, naringenin and silybin were found to suppress activities of *EGFP* and *Rluc* marker genes expressed by the CHIKV replicon. In a concomitant screen against Semliki Forest virus (SFV), their anti-alphaviral activity was confirmed and several additional inhibitors of SFV with IC_50_ values between 0.4 and 24 µM were identified. Chlorpromazine and five other compounds with a 10*H*-phenothiazinyl structure were shown to inhibit SFV entry using a novel entry assay based on a temperature-sensitive SFV mutant. These compounds also reduced SFV and Sindbis virus-induced cytopathic effect and inhibited SFV virion production in virus yield experiments. Finally, antiviral effects of selected compounds were confirmed using infectious CHIKV. In summary, the presented approach for discovering alphaviral inhibitors enabled us to identify potential lead structures for the development of alphavirus entry and replication phase inhibitors as well as demonstrated the usefulness of CHIKV replicon and SFV as biosafe surrogate models for anti-CHIKV screening.

## Introduction

Arthropod-borne viruses (arboviruses) are currently regarded as re-emerging threats for health and well being in tropical regions and, as a consequence of vector spread, also in more temperate areas [1]. Factors including population growth and urbanization, increased travel, ignorance of control methods for mosquito vectors and climate change have been considered to contribute to the increased risk of diseases caused by arboviruses, many of which lack efficient antiviral therapies or vaccination [2].

Currently recognized arboviruses are single-stranded RNA viruses in the families *Flaviviridae*, *Togaviridae*, *Bunyaviridae* and *Rhabdoviridae*. Alphaviruses (within family *Togaviridae*) have enveloped virions of icosahedral symmetry and an RNA genome of approximately 11.5 kb in size, which contains two open reading frames [3]. These viruses enter their host cells via receptor-mediated endocytosis. After fusion of the virus envelope with endosomal membranes, the nucleocapsid is disassembled to release the 5′ capped positive stranded RNA genome. Immediate translation of the RNA yields polyprotein P1234, the precursor of virus nonstructural (ns) proteins nsP1-nsP4. Early processing of the P1234 polyprotein releases the core polymerase subunit nsP4. NsP4 together with the intermediate cleavage product P123 form the negative strand RNA polymerase complex, producing the templates for further positive strand synthesis. Processing of P123 results in the release of individual ns-proteins nsP1-nsP3, and switches the RNA synthesis to production of RNA with positive polarity. In addition to the genomic RNA coding for ns-proteins, a subgenomic (sg) RNA is produced by internal initiation from the negative strand template, allowing translation of virus structural proteins. Nucleocapsids are assembled in the cytoplasm, and they recognize the virus envelope proteins at the plasma membrane, where budding occurs.

The clinical importance of alphaviruses has been underscored by the recent epidemic outbreaks of Chikungunya virus (CHIKV) in different sites around the Indian Ocean, including La Réunion and other islands, India, and South-East Asia [4], [5]. The epidemic from 2005 to late 2007 has been estimated to include more than 6 million cases. Furthermore, an outbreak of approximately 200 confirmed cases took place in Italy, and imported cases in travellers returning from endemic areas were reported in several European countries, USA, Canada and Australia [6], [7]. The ecology of arboviral species typically relies on the amplification of viral pools in wild rodents or birds and large outbreaks have been associated with nearby forest or wetland to allow such zoonotic cycles [2]. However, the rise of mosquito species adapted to urban environments (e.g. *Aedes albopictus*) has changed the pattern, and the recent CHIKV epidemic is thought to have arisen from direct human-to-human transmissions by feeding mosquitoes [5].

Clinical CHIKV infection is characterized by acute, febrile illness and high viremia (up to 10^10^ copies/ml of viral genomes in serum) that lasts for 3–10 days [8]. The clinical symptoms of CHIKV and other Old World alphavirus infections include high fever and other flu-like symptoms resulting from the proinflammatory cytokine response to virus, maculopapular rash and related skin disorders, as well as gastrointestinal problems such as nausea and vomiting. Approximately 10–30% of the patients suffer from symptoms of connective tissues, mainly myopathy and arthralgia. The joint pain resembles rheumatoid arthritis as it is most intense in the small joints of extremities, and follow-up studies of patients have indicated that these symptoms may persist for several months [9]. The role of the proinflammatory response has been connected also to the muscle and joint manifestations [10], and these symptomatic tissues have also been shown to be the sites of *in vivo* virus replication [11]–[13]. In the recent CHIKV outbreak, a high proportion of neurological symptoms were observed in neonates and small children infected with CHIKV [14]. Encephalitis and meningoencephalitis were observed in half of the infected small children, and persistent disabilities are estimated in 10–20% of these cases.

The medical treatment of alphavirus infections relies on symptomatic relief, as no effective treatment is available to affect virus replication. During the 2006 La Réunion outbreak, a double-blind, randomized clinical trial was conducted to evaluate the efficacy of chloroquine in acute CHIKV viremia, but the study failed to show any benefits in terms of the duration of viremia or the severity and duration of clinical symptoms [15]. Previous reports on alphavirus inhibitors are scarce and involve mainly broad-spectrum antiviral agents targeting cellular enzymes such as inositol monophosphate dehydrogenase, S-adenosyl homocysteine hydrolase and orotidine 5′-phosphate decarboxylase [16]–[18]. Many of these compounds are limited by their narrow therapeutic index or immunomodulatory effects that are considered unfavorable for the treatment of clinical infection.

The discovery of CHIKV inhibitors is hampered due to the requirement for biosafety level 3 (BSL-3) handling. To overcome this issue, we report in this study the generation of a stable BHK cell line harboring non-cytotoxic CHIKV replicon and the adaptation of this cell line as a screening tool for identification of alphavirus inhibitors. A focused library of 123 natural and 233 pharmaceutical compounds was screened against the CHIKV replicon, as well as against infectious Semliki Forest virus (SFV). Activity of selected compounds was also confirmed using infectious CHIKV. Furthermore, a virus entry inhibition assay was established based on a temperature-sensitive (ts) SFV mutant SFVts9. These experiments revealed the inhibition of CHIKV and SFV replication by 5,7-dihydroxyflavones and the inhibitory effect of 10*H*-phenothiazines on alphavirus entry. The approach used in this study demonstrates the benefits and suitability of using CHIKV replicon and SFV as biosafe surrogate models for anti-CHIKV screening.

## Results

### Generation of a stable CHIKV replicon cell line

**Figure 1:**
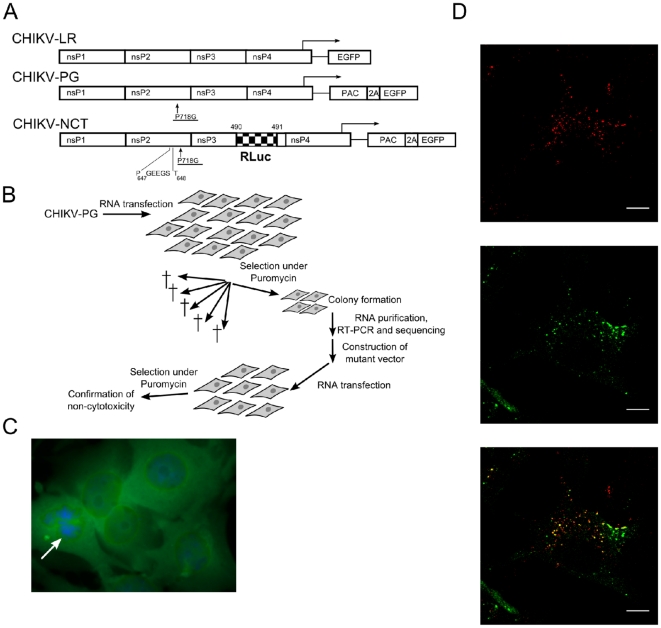
Construction and characterization of a stable BHK cell line carrying CHIKV-replicon. A) Schematic representation of the used CHIKV replicons (numbers and symbols are explained in the text). B) The process leading to selection of non-cytotoxic (NCT) CHIKV replicons, identification of mutations associated with the NCT phenotype and confirmation of their phenotypes. C) Phenotype of BHK-CHIKV-NCT cells; green fluorescence is caused by EGFP expression. Arrow indicates a cell in the process of division. D) Immunofluorescence images of BHK-CHIKV-NCT cells stained with anti-dsRNA (top), anti-SFV nsP3 (middle) and co-staining with anti-dsRNA and anti-SFV nsP3 (bottom). A representative optical slice from the middle of the cell is shown. Scale bar is 10 µm.

The most prominent human pathogen among the Old World alphaviruses, CHIKV is an infectious agent that in most countries requires handling in BSL-3 facilities. Our aim was to establish a more screening-friendly assay system to identify inhibitors of CHIKV replication (i.e., a BHK-based cell line containing persistently replicating CHIKV replicon RNA). A selection marker (puromycin acetyltransferase, Pac) and two reporter genes (*Renilla* luciferase, *Rluc* and *EGFP*) were inserted into the sequence of CHIKV-LR replicon originating from an isolate from La Réunion [19] (Fig. 1A). To reduce the cytotoxicity of the wild-type CHIKV-LR replicon, a Pro718 to Gly substitution in nsP2, previously shown to reduce the cytotoxicity of SFV and SINV vectors [20]–[22], was introduced into the protease-encoding section to yield CHIKV-PG. Without this mutation, all cells transfected with transcripts from such vectors invariably died (data not shown).

The selection process is illustrated in Fig. 1B. BHK cells transfected with *in vitro* transcripts from the CHIKV-PG vector were plated and puromycin selection (5 µg/ml) was applied starting from 16 h post-transfection. Most of the cells died within four days, but the remaining cells (roughly estimated as one out of 1×10^5^ transfected cells) expanded to cell clones which were transferred to separate plates and subsequently expanded to cell lines under continuous puromycin selection. The total RNA from 12 independent cell lines was purified and the regions corresponding to CHIKV nsP2 were amplified by RT-PCR and sequenced to identify mutations responsible for the non-cytotoxic phenotype of the resulting replicon. Each of the identified mutations was introduced into the CHIKV-PG vector and the BHK-21 cells, transfected with such mutant replicons, were subjected to cell viability assays (data not shown). Based on these experiments, a single mutation representing an insertion of five amino acid residues (GEEGS; sequence of the corresponding insert in the replicon RNA was GGG GAG GAA GGG AGU) between residues 647 and 648 of CHIKV nsP2 was chosen. The insertion lay at a site where a nuclear localization signal has been discovered in SFV nsP2 [23]. This mutation was incorporated into CHIKV-PG, together with an *Rluc* marker fused with nsP3, to obtain CHIKV-NCT replicon vector (Fig. 1A). BHK cells transfected with this replicon were viable under continuous puromycin selection and were designated as BHK-CHIKV-NCT cells.

### Characterization of the BHK-CHIKV-NCT cell line

The appearance and speed of division of BHK-CHIKV-NCT cells were similar to those of parental BHK cells, but these cells were resistant to puromycin and expressed high levels of *EGFP* (Fig. 1C) and *Rluc* markers throughout at least 20 passages. In immunofluorescence studies, the BHK-CHIKV-NCT cells were positive for double-stranded RNA (dsRNA) (Fig. 1D, top). The cells could also be stained by polyclonal antibodies against SFV nsP3 (Fig. 1D, middle), showing the cross-reactivity of these antibodies with CHIKV nsP3. NsP3 and dsRNA were co-localized in the replicon containing cells (Fig. 1D, bottom), indicating the presence of replication complexes with a typical alphaviral localization [24] in the perinuclear region of the cells and, in minor quantities, at the plasma membrane.

**Figure 2:**
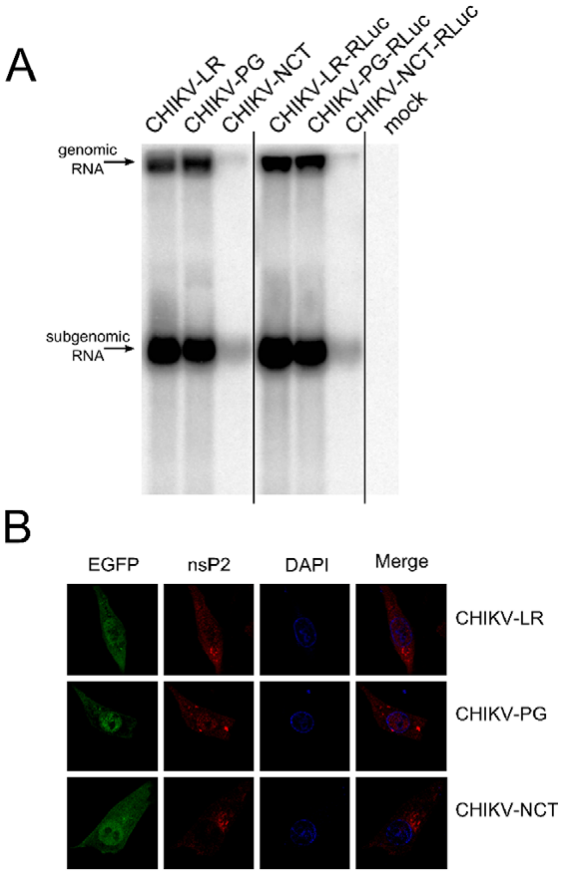
Effects of adaptive mutations on the CHIKV replicon. A) Effects of the PG and NCT mutations on the accumulation of positive-strand replicon and corresponding sgRNAs in cells transfected with *in vitro* transcripts of CHIKV-LR, CHIKV-PG, CHIKV-NCT and their variants containing the *Rluc* marker in the nsP3 region. Total RNAs extracted from transfected cells at 16 h post-transfection; 10 µg aliquot from each sample was separated by electrophoresis in formaldehyde gel and analyzed by Northern blotting. The constructs are shown at the top; positions of replicon and sgRNAs are indicated with arrows. “Mock” indicates RNAs from mock-transfected control cells. B) Immunofluorescence analysis of nsP2 localization in cells transfected with *in vitro* transcripts of CHIKV-LR or CHIKV-PG at 8 h post-infection or with in *vitro* transcripts of CHIKV-NCT at 16 h post-infection. Cells were fixed and stained with anti-nsP2 and DAPI; EGFP was detected by fluorescence. Merge indicates co-staining of DAPI and nsP2.

To characterize the phenotypic changes caused by mutations in the nsP2 region, the total RNA from BHK cells transfected with CHIKV-LR (wild type), CHIKV-PG and CHIKV-NCT replicons was analyzed using Northern blotting. This assay revealed that, in contrast to SINV [21] and SFV [22], the introduction of the PG-mutation into the CHIKV replicon led only to a slight reduction of the accumulation of replicon and corresponding sgRNAs. However, the levels of both replicon and sgRNAs of CHIKV-NCT were severely reduced (Fig. 2A). At the same time the levels of marker expression (both *EGFP* and *Rluc*) in CHIKV-NCT transfected cells were comparable with those achieved by the use of CHIKV-LR or CHIKV-PG replicons (before cell death occurred). The discrepancy between the levels of viral RNAs and their translation products could be explained by the lack of translational shutdown in the cells transfected with CHIKV-NCT, which greatly enhances translation of both genomic RNA and sgRNA, lacking the region corresponding to the translational enhancer sequence of Sindbis virus (SINV). A similar phenomenon has been previously described for related SFV replicons [23], [25]. In addition, this analysis demonstrated that the insertion of the *Rluc* marker into the nsP3 region had no detectable effect on the replication and transcription of corresponding replicons (Fig. 2A).

As the nuclear localization of nsP2 has been shown to affect the cytotoxic properties of both SFV and replicons derived from it [22], [23], the effects of the introduced mutations on the subcellular localization of nsP2 of CHIKV were analyzed by immunofluorescence. This analysis revealed that at 8 h post-transfection with CHIKV-LR RNA, a fraction of nsP2 was localized in the nucleus of cells (Fig. 2B). Consistent with data reported for SFV replicons, the presence of the PG-mutation resulted in slightly increased nuclear localization of nsP2 (Fig. 2B), while in cells transfected with CHIKV-NCT replicons, nsP2 was largely, but not completely, excluded from the nuclei (Fig. 2B). It should be noted that some variation in nsP2 localization between individual transfected cells was also observed for each of the analyzed constructs (data not shown).

The replicon present in BHK-CHIKV-NCT cells contains two reporter genes, *Rluc* fused with CHIKV nsP3 and *EGFP*, which is produced as a fusion protein with Pac under the sg-promoter (Fig. 1A). *EGFP* is processed away from Pac by Foot-and-Mouth Disease Virus (FMDV) 2A autoprotease sequence and is released into the cytoplasm. The BHK-CHIKV-NCT cells had intense luminescent and fluorescent signals when detected with a plate reader in 96-well plate format, showing signal to background (S/B) ratios of approximately 340 for the luminescent and approximately 60 for the fluorescent signal when the native BHK cells were used as background.

For all experiments with antiviral compounds, puromycin was excluded from the assay media to avoid puromycin-induced toxicity as a response to suppression of Pac expression linked to the replicon expression levels. The replicon responded to the reference compounds used in the study in the low micromolar range. The dose-response curves for ribavirin, mycophenolic acid and 6-azauridine determined with both *EGFP* and *Rluc* signals revealed sigmoidal, dose-dependent reduction in both marker levels. The 50% inhibitory concentrations (IC**_50_**) were approximately 1 µM for mycophenolic acid and 6-azauridine with both reporter genes, and 8.8 µM for ribavirin using *EGFP* and 25.4 µM using *Rluc* (Table 1). Chloroquine showed no suppression of replicon propagation, which was expected because of its mode of action. It inhibits several viruses by blocking pH-dependent steps in virus entry and maturation, neither of which are present in the used replicon systems [26], [27]. Furthermore, the IC_50_ values of ribavirin and mycophenolic acid were increased by at least two orders of magnitude when the cultures were supplemented with 50 µg/ml guanosine (data not shown). This result indicated that the observed suppression of *EGFP* and *Rluc* was a consequence of cellular guanosine depletion, a generally accepted mode of action for ribavirin and mycophenolic acid [28], [29].

**Table 1:** Inhibition of CHIKV replicon in BHK-CHIKV-NCT cells by hit and reference compounds.

Compound	*EGFP* a IC_50_(µM)	*Rluc* aIC_50_(µM)	Cell viability^b^ IC_50_ (µM)
**NC compounds**			
Apigenin	22.5	28.3	>200
Chrysin	46.8	50.2	>200
Naringenin	25.8	30.0	122.1
Silybin	71.1	59.8	>200
**PC compounds**			
Prothipendyl	135.0	93.3	185.6
**Reference compounds**			
Ribavirin	8.8	25.4	>200
Mycophenolic acid	1.5	4.1	>200
6-Azauridine	2.4	3.1	>200
3′-NH_2_-3′-deoxyadenosine	34.0	62.4	187.1

^*a*^IC_50_ values for suppression of CHIKV replicon were determined by exposing the replicon cell line to test compounds at various concentrations from 200 µM to 10 nM for 48 h.

^*b*^Cell viability IC_50_ values were determined by ATP assay after 48 h exposure of BHK-CHIKV-NCT cells. All results represent the mean values from two individual experiments both run in triplicate (CV ranged from 4.9 to 13.7% in the CHIKV replicon assay). NC = natural compounds, PC = pharmaceutical compounds.

### Screening for CHIKV replication inhibitors

After characterization and adaptation for screening, the BHK-CHIKV-NCT cell line was used for screening a total of 356 compounds, including 123 natural compounds and 233 clinically approved drugs and other pharmaceutical compounds. These libraries were selected due to the following reasons. First, natural compounds, such as flavonoids and coumarins, are present in herbal medicines typically used in the endemic areas of CHIKV and therefore finding a potential inhibitor among these natural compounds may provide evidence for the potential use of certain herbal medicines to treat CHIKV infections. Second, by screening a collection of known drugs instead of a random chemical library, it is possible to focus the assaying on compounds that are already shown to be clinically approved. After 48 h exposure of the replicon-containing cell line to 50 µM compounds, *EGFP* levels of the cell cultures were read as the endpoint for the primary screen. The hit limit in the screen was set as >75% reduction of the *EGFP* signal, and the antiviral activity of all compounds scoring as actives was confirmed in a replicate experiment determining both *EGFP* and *Rluc* marker levels. Dose-dependent suppression of the marker genes included in the replicon vector after 48 h exposure was observed for natural compounds apigenin, chrysin, naringenin and silybin, and for one pharmaceutical compound, prothipendyl. The IC**_50_** values of the four natural compounds and prothipendyl are presented in Table 1. As a measure of selectivity, the viability of BHK-CHIKV-NCT cells after 48-h exposure with hit compound concentrations of up to 200 µM was determined. As indicated in Table 1, all compounds except naringenin (cell viability IC**_50_** 103.5 µM) and prothipendyl (cell viability IC**_50_** 185.6 µM) were well tolerated by the BHK-CHIKV-NCT cells at the highest concentration used (200 µM).

### Screening against infectious SFV

Using a previously described antiviral assay based on an SFV strain with *Rluc* inserted in between nsP3 and nsP4 (SFV-*Rluc*) [30], the same set of 356 compounds was assayed against SFV, an alphavirus closely related to CHIKV. BHK cells were infected with SFV-*Rluc* (MOI 0.001), the compounds were added at 50 µM concentration simultaneously with the virus inocula, and the marker gene expression level was determined at 14 h post-infection. Similarly to the CHIKV replicon screen, the hit limit of >75% reduction of *Rluc* marker level was applied. After excluding obviously toxic compounds (100% cell death detected by visual inspection of non-infected cells under light microscope), 14 natural compounds and 12 pharmaceutical compounds were identified as screening hits against SFV-*Rluc* (Table 2). Consistent with the CHIKV replicon screen, all five chemical agents identified as CHIKV replicon inhibitors were found to inhibit SFV infection as well. A complete list of primary screening results can be found in Table S1.

**Table 2:** Compounds that inhibit SFV replication in the SFV-*Rluc* screening assay.

Compound	SFV^a^I C_50_ (µM)	Cell viability^b^IC_50_ (µM)	SI^c^
**NC compounds**			
Alphanaphtoflavone	24.1	>200	>8.3
Apigenin	20.6	>200	>9.7
Bergapten	8.1	>200	>24.6
Chrysin	13.7	>200	>14.6
Coumarin 30	0.4	92.4	231
7-Diethylamino-3-thenoylcoumarin	5.1	39.1	7.7
4-Hydroxyacetophenone	21.1	164.0	7.8
Methyl umbelliferone	18.8	179.8	9.9
Naringenin	2.2	94.1	42.8
Propyl gallate	17.9	102.8	5.8
Protocatechuic acid	8.0	>200	>25.0
Pyrogallol	18.7	>200	>10.7
Quercitrin	23.7	>200	>8.4
Silybin	16.4	>200	>12.2
**PC compounds**			
Chlorpromazine	15.7	67.3	4.5
Doxepin	14.5	>200	>13.8
Ethopropazine	16.0	166.9	10.4
17-Ethinylestradiol	9.5	>200	>21.1
Menadione	7.8	21.9	2.8
Methdilazine	11.3	63.8	5.6
Nadoxolol	16.4	>200	>12.2
Opipramol	19.7	>200	>10.2
Perphenazine	25.1	155.0	6.2
Prothipendyl	8.2	>200	>24.4
Thiethylperazine	15.0	83.1	5.5
Thioridazine	14.9	179.4	12.0
**Reference compounds**			
6-Azauridine	>200	>200	-
Chloroquine	13.4	>200	>14.9
3′-NH_2_-3′-deoxyadenosine	16.2	173.8	10.7
Mycophenolic acid	121.1	>200	>1.7
Ribavirin	95.1	>200	>2.1

^*a*^IC_50_ values for SFV replication were determined using SFV-*Rluc* infection (MOI 0.001 in BHK cells and detection at 14 h post-infection).

^*b*^Cell viability IC_50_ values were determined by ATP assay after 48 h exposure of BHK cells.

^*c*^Selectivity indices (SI) were calculated as the ratio of the two values. All results represent the mean values of two individual experiments both run in triplicate (CV ranged from 8.1 to 21.3% in the SFV-*Rluc* experiments). NC = natural compounds, PC = pharmaceutical compounds.

The screening hits were further analyzed by dose-response experiments. Cell viability IC**_50_** values were determined as described above and selectivity indices were calculated for each compound as the ratio of cell viability and antiviral IC**_50_**. Table 2 presents antiviral and cell viability IC_50_ values, and selectivity indices for all anti-SFV hit compounds. The results obtained with positive controls mycophenolic acid, 6-azauridine, chloroquine and 3′-amino-3′-deoxyadenosine are also included in Table 2. Several anti-SFV screening hits exhibited antiviral IC_50_ values in the low micromolar range. For example, a synthetic coumarin derivative, coumarin 30, had an IC_50_ value of 0.4 µM against SFV and a selectivity index of 308, whereas one of the flavonoids, naringenin, had an IC_50_ value of 2.2 µM and a selectivity index of 47.

### Inhibition of virus-induced CPE and SFV yield

A selectivity index >10 was set as a threshold for selecting anti-SFV hit compounds for characterization by other assays, yielding 8 natural compounds and 7 pharmaceutical compounds. Concerning these 15 selected compounds, studies were extended to assay their capacity to reduce virus-induced cytopathic effect (CPE) and to measure the inhibition of virus production. Besides SFV, a distantly related member of the alphavirus genus, SINV, was included in the CPE reduction studies as well. Table 3 lists the IC_50_ values of these compounds in the CPE reduction assay for both SFV and SINV, detected at 22 h and 24 h post-infection (SFV and SINV, respectively; MOI 0.01) using WST-1 tetrazolium salt to quantify cell viability. Although two natural compounds and one pharmaceutical compound (protocatechuic acid, pyrogallol and 17-ethinylestradiol) failed to inhibit the CPE induced by SFV or SINV (determined with a cut-off value >25% higher cell viability than in non-treated infection), all three compounds showed reproducible inhibition in the primary screening assay using SFV-*Rluc*. However, the lack of activity in CPE reduction assay was consistent with the results from virus production experiments, in which none of the three compounds reduced SFV yields (see below). The remaining compounds included in the experiments showed consistent results when compared to the SFV-*Rluc* assay, exhibiting IC_50_ values in a similar range as observed with the reporter gene assay. The reference compounds ribavirin and mycophenolic acid performed better in the CPE assay than in the screening assay: ribavirin had an IC_50_ value of 28.1 µM against SFV and 51.8 µM against SINV (compared to 95.1 µM in the screening assay). In the case of mycophenolic acid, the values were 39.0 µM and 44.4 µM for SFV and SINV in the CPE reduction, respectively, and 121.1 µM in the reporter gene assay. Chloroquine, 3′-amino-3′-deoxyadenosine and 6-azauridine did not show similar shifts in IC_50_ values between the two assays, resembling the newly identified antiviral hit compounds in this respect.

**Table 3:** Inhibition of SFV and SINV replication by selected hit compounds measured by CPE reduction and virus production assays.

Compound	SFV^a^ IC_50_ (µM)	SINV^a^ IC_50_ (µM)	SFV yield^c^ (PFU/ml)
**NC compounds**			
Apigenin	4.4	7.2	6.7×10^7^
Bergapten	9.2	16.2	1.2×10^8^
Chrysin	22.9	43.3	2.9×10^8^
Coumarin 30	5.5	11.8	5.0×10^8^
Naringenin	19.3	21.2	8.2×10^7^
Protocatechuic acid	>200	>200	2.4×10^9^
Pyrogallol	>200	>200	1.1×10^9^
Silybin	9.6	22.6	3.7×10^8^
**PC compounds**			
Doxepin	50.7	41.1	3.6×10^8^
Ethopropazine	17.1	21.4	8.1×10^8^
17-Ethinylestradiol	63.0	56.8	9.0×10^8^
Nadoxolol	15.2	8.2	9.2×10^7^
Opipramol	25.0	31.0	2.5×10^8^
Prothipendyl	34.2	46.5	4.0×10^8^
Thioridazine	19.3	37.3	6.8×10^8^
**Reference compounds**			
6-Azauridine	>200	>200	9.1×10^7^
Chloroquine	8.2	11.3	3.3×10^7^
3′-NH_2_-3′-deoxyadenosine	17.5	23.4	2.7×10^7^
Mycophenolic acid	39.0	44.4	1.2×10^8^
Ribavirin	28.1	51.8	2.1×10^8^

^*a*^IC_50_ values against SFV and SINV were determined in dose-response experiments using an assay for the reduction of cytopathic effect. A concentration range from 200 µM to 0.1 µM was applied for each compound. The results represent the mean values from two individual experiments, both run in triplicate (CV ranged from 9.9 to 18.6%).

^*c*^The effect of hit compounds on SFV yield was analyzed by determining SFV titers of infection samples grown in the presence of 50 µM hit compound. The untreated control sample had an SFV titer of 1.4×10^9^ PFU/ml. NC = natural compounds, PC = pharmaceutical compounds.

The rightmost column in Table 3 lists the SFV yields in a virus production assay, where BHK cells were infected with SFV (MOI 0.01) in the presence of 50 µM compounds. After 16 h, the infection media were collected and SFV titers in each sample were determined by plaque titration. Untreated control infection yielded an SFV titer of 1.4×10^9^ PFU/ml under these conditions, while ribavirin and mycophenolic acid decreased the virus titer by approximately one order of magnitude, and chloroquine and 3′-amino-3′-deoxyadenosine by two orders of magnitude. Among the natural compound hits, apigenin and naringenin showed the greatest decrease in SFV yield, both in the same range as reference compounds used in the study (apigenin-treated infection yielded an SFV titer of 6.7×10^7^ PFU/ml and naringenin-treated culture, 8.2×10^7^ PFU/ml). Among the pharmaceutical compounds, best results were achieved with nadoxolol (SFV titer 9.2×10^7^ PFU/ml) and opipramol (SFV titer 2.5×10^8^ PFU/ml).

### SFV entry inhibition

Because the SFV screen revealed several hits not identified as CHIKV replication inhibitors in the replicon assay, virus entry as a potential target step for the anti-SFV activity was studied by SFV-*Rluc* with a G389R point mutation in nsP2 (SFVts9-*Rluc*). Based on our earlier work, this mutation causes defects in the NTPase and RNA triphosphatase (RTPase) enzymatic activities of the N-terminal domain of nsP2 and is accompanied by site-specific defects in P1234 polyprotein processing [31], [32]. These defects result in a ts-phenotype, characterized by severe defects in RNA replication at an elevated temperature (0.5% of RNA replication compared to wild-type SFV at 39°C), but replication levels are comparable to the wild-type virus when grown at the permissive temperature of 28°C. Because the virus is unable to multiply its RNA genome at 39°C, all *Rluc* accumulating in BHK cells after infection at the restrictive temperature results from the translation of the initial RNA strands upon virus entry. This feature was used to set up an assay to evaluate the effects of the hit compounds on SFV entry by detecting *Rluc* in cell culture lysates infected with SFVts9-*Rluc* at 39°C.

**Figure 3:**
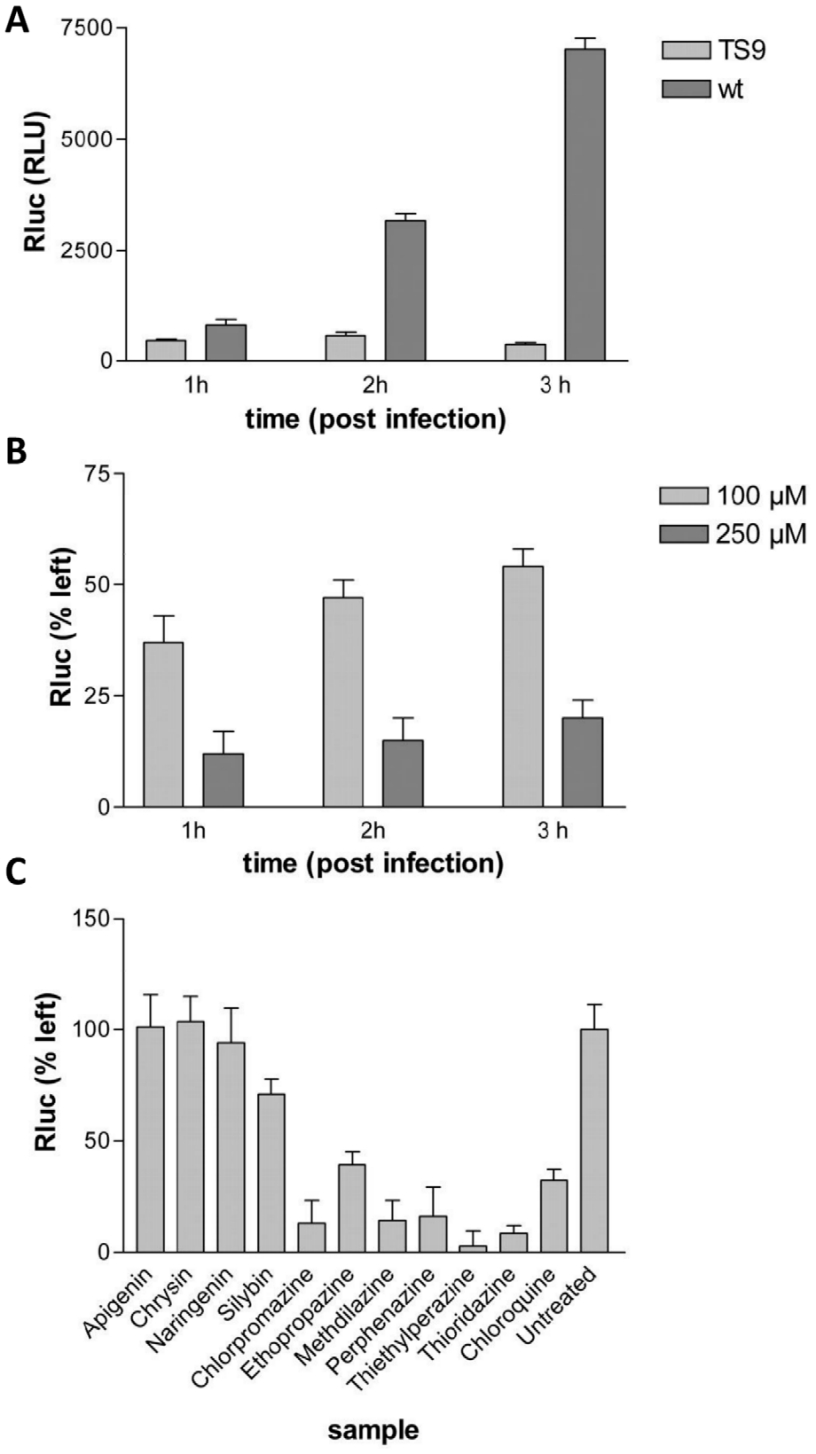
Virus entry assays with SFVts9-*Rluc*. A) The temperature-sensitive phenotype of SFVts9-*Rluc* as measured by *Rluc* activity of infected cell lysates at 1 h, 2 h and 3 h post-infection at 39°C. B) The effect of chloroquine on *Rluc* signals of SFVts9-*Rluc* at 39°C. C) Effects of 5,7-dihydroxyflavones and 10*H*-phenothiazines on the accumulation of *Rluc* in cells infected with SFVts9-*Rluc* at 39°C. Selected examples of results of hit compounds in SFV entry inhibition assay. The cell cultures were treated with 100 µM compounds and *Rluc* levels were determined 1 h post-infection. All results represent the average of three replicates.

The ts-phenotype of the propagated SFVts9-*Rluc* virus was confirmed in experiments performed at 39°C using wild-type SFV as a control virus (Fig. 3A). At 28°C, the *Rluc* counts of SFVts9-*Rluc* were higher and increased with time (data not shown). Chloroquine, a lysosomotrophic weak base with well-characterized inhibitory effects on the entry of SFV and several other enveloped viruses, was assayed in the system to define the sensitivity towards chemical agents acting as entry inhibitors. The response to chloroquine was measured at concentrations of 100 and 250 µM and showed a dose-dependent inhibition of *Rluc* signal (Fig. 3B). At lower concentrations of the drug, virus entry may slowly continue at extended time points, leading to increases in the signal. Based on this finding and the fact that without the drug, maximal signal was reached in 1 h for SFVts9-*Rluc* (Fig. 3A), the 1-h end point was selected for the library compound experiments.

To assay the hit compounds listed in Table 2 with the entry inhibition assay, the compounds were added at 100 µM concentration simultaneously with SFVts9 infection, and *Rluc* activities were measured in lysates collected at 1 h post-infection. Fig. 3C presents selected examples of the results with the hit compounds. Six pharmaceutical compounds (chlorpromazine, ethopropazine, methdilazine, perphenazine, thiethylphenazine and thioridazine) decreased the *Rluc* activity, indicating that these six compounds sharing a common core structure of 10*H*-phenothiazine inhibited SFV entry. None of the other compounds, including the flavonoids apigenin, chrysin, naringenin and silybin, inhibited SFV entry in the assay.

### Inhibitory activities of selected hit compounds against infectious CHIKV

As demonstrated above, two surrogate models of CHIKV, the BHK-CHIKV-NCT replicon cell line and related infectious SFV can be used for screening of potential inhibitors. To validate the selected hits, the recombinant CHIKV-LR virus with the *Rluc* marker fused with nsP3 in the same way as in CHIKV-NCT replicon (Fig. 1A), was constructed. The virus, designated as CHIKV-*Rluc*, was found to be genetically stable and was used for subsequent assays. In total, twelve compounds that were identified in the screens described above were analyzed. These compounds comprised five compounds originating from BHK-CHIKV-NCT-based screens (Table 1), the most potent anti-SFV hit coumarin 30 and six SFV entry inhibitors (Fig. S1). 6-Azauridine was included as a reference compound. The assay was carried out as described for SFV-*Rluc* with modifications indicated in the Materials and Methods section. Using this assay, 6-azauridine was found to inhibit CHIKV-*Rluc* with an IC_50_ value 29.7 µM. This is slightly higher than previously reported for CHIKV (0.2 µg/ml; approximately 0.8 µM, ref. 16), which is in line with our earlier observations that *Rluc*-based antiviral screening assays typically yield slightly lower potency estimates than CPE or RNA labeling assays. The IC_50_ value for 6-azauridine in the CHIKV-*Rluc* based assay was approximately ten times higher than observed in the replicon-based assay and the same tendency was reproducibly observed for all of the other compounds tested. Aside from this trend, the results obtained with CHIKV-*Rluc* were consistent with those obtained using the surrogate systems. Similarly to the case of SFV-*Rluc* (Table 2), coumarin 30 was found to be the most potent inhibitor of CHIKV-*Rluc* as well (IC_50_ value of 6.4 µM). All compounds that inhibited replication of CHIKV-NCT also inhibited infection of CHIKV-*Rluc*: IC_50_ of 70.8 µM for apigenin, 126.6 µM for chrysin, 118.4 µM for naringenin, 92.3 µM for silybin and 97.3 µM for prothipendyl. Thus, apigenin was again identified as the most potent inhibitor from this group of compounds; however, the IC_50_ values for this compound and the other compounds (except prothipendyl) were 2–3 fold higher than those observed in the BHK-CHIKV-NCT cell-based assay. All compounds identified as inhibitors of SFV entry also inhibited CHIKV-*Rluc* infection. When compared to the SFV-*Rluc* based screening results (Table 2), the entry inhibitors showed similar potencies against the CHIKV-*Rluc*; however, the IC_50_ values determined using the CHIKV-*Rluc* were higher (39.4 µM for chlorpromazine, 48.1 µM for perphenazine, 61.5 µM for ethopropazine, 63.8 µM for thietylperazine, 71.5 µM for thioridazine and 84.5 µM for methdilazine) than the range of 11.3 µM–25.1 µM. Thus, all compounds tested using infectious CHIKV were confirmed to inhibit its infection, indicating that the use of a combination of surrogate screening systems did not result in false-positive hits.

## Discussion

The current study presents the development of a novel tool for bioactivity screening and molecular studies on CHIKV: a stable BHK cell line harboring CHIKV replicon (BHK-CHIKV-NCT). Phenotypic antiviral assays with infectious CHIKV cannot be performed in most screening facilities due to BSL-3 requirements, and thus far, only few studies have validated individual target proteins as potential sites for medical intervention for CHIKV [33]. Given the shortage in screening approaches with isolated target proteins, CHIKV replicon cell lines offer a screening-friendly approach in this respect. The BHK-CHIKV-NCT cell line, persistently expressing a CHIKV replicon including Pac, *EGFP* and *Rluc*, was found to grow as fast as the native BHK cell line, to be stable for at least 20 passages, to express high levels of both markers and to respond to known alphavirus replication inhibitors in a concentration range comparable to previous publications. The hits found using the BHK-CHIKV-NCT cell line were confirmed by another novel tool, infectious CHIKV-*Rluc*, indicating that the different nature of the screening system and compromised replication of CHIKV-NCT replicon did not result in selection of false-positive inhibitor candidates.

Within the *Alphavirus* genus, CHIKV and SFV belong to the same antigenic serocomplex and are considered phylogenetically closely related [34]. The well-conserved nature of the replicase proteins within the two virus species was also demonstrated by the recognition of CHIKV nsP3 by the anti-SFV nsP3 antibody. The high level of correlation between the screening data with the two focused libraries against the BHK-CHIKV-NCT replicon cell line and the SFV also provides proof that SFV can be used as a reliable surrogate virus species for the identification of broad-spectrum antiviral agents against CHIKV and other alphaviruses. However, in the case of any user-friendly surrogate system, the possibility of false-negative and false-positive hits does exist. Therefore, the verification of the hits using infectious CHIKV represents an important proof for the applicability (and practical value) of these surrogate systems, as shown in this study using infectious CHIKV-*Rluc*.

In vertebrate cells, wild-type alphaviruses cause an acute infection characterized by CPE, a severe decrease in host cell viability typically occurring within 24 h post-infection [35]. In the case of Old World alphaviruses, CPE is induced at least in part by nsP2, which is responsible for host cell transcriptional silencing via an unidentified interaction of nsP2 with host cell factors, among other actions [36], [37]. Thus, wild-type replicons of Old World alphaviruses (e.g., SFV, SINV and CHIKV) cannot be used to generate stable cell lines. Achieving this aim requires modification of nsP2 to reduce the cytotoxicity of the replicon to the host cells. These cell lines have been previously obtained for SINV and SFV [25], [39] and used as tools for recombinant protein expression or as tools for the study of protein function [25], [37]–[39]. However, because these viruses do not represent significant human pathogens, the use of these cell lines for antiviral drug development has not been reported.

The requirements to achieve a non-cytotoxic phenotype of replicons for different Old World alphaviruses are not identical. A single point mutation [38], [39] or a five-amino acid insertion in certain positions of nsP2 region [40] are both sufficient to create non-cytotoxic SINV replicon and to obtain a stable BHK cell line carrying such a replicon. In contrast to SINV, the P718G (corresponding to P726G in SINV) or P718T mutation alone only reduced the cytotoxicity of SFV replicons when applied individually but was not sufficient to make them non-cytotoxic [22], [25]. In the case of the SFV replicon with the P718T mutation, it was shown that an additional R649H mutation, obtained during puromycin selection, was required to achieve a truly non-cytotoxic phenotype. Furthermore, it has been reported that the use of the same selection procedure for CHIKV replicon with a P718S mutation did not result in selection of any non-cytotoxic replicons [41]. Because the approach successfully used in this study (Fig. 1B) differed only in the mutation used in the original replicon (P718G instead of P718S), one may speculate that the requirements for a non-cytotoxic phenotype of CHIKV replicons are slightly different and may be stricter than in the case of SFV. This assumption was supported by the observation that the P718G mutation had only a minor effect on RNA replication (Fig 2A) and cytotoxicity of CHIKV replicons, while analogous the mutation in the SFV replicon caused a much more prominent effect [22].

The reduction of replication/transcription is a common theme for all non-cytotoxic replicons of Old World alphaviruses [21], [22], [25], [38], and therefore, it is not surprising that the CHIKV-NCT replicon clearly differed from the parental CHIKV-LR replicon in reduced synthesis of viral positive-strand RNAs (Fig. 2A). This finding is consistent with the data previously reported for SFV vectors with reduced cytotoxicity [22] and indicates that reduced replication is likely to represent one of the factors contributing to the non-cytotoxic nature of CHIKV-NCT replicons. In contrast, the significance of the nuclear location of nsP2 for the non-cytotoxic phenotype is less clear [40]. The PRRRV sequence, shown to function as a nuclear localization signal in SFV nsP2 [23], is not well conserved within alphaviruses. Additionally, for SINV nsP2, the nuclear transport of nsP2 does not solely depend on the presence of SV40-type nuclear localization signals [40]. In the region corresponding to the SFV PRRRV sequence, the CHIKV nsP2 contains a PTKRV sequence not predicted to represent a nuclear localization signal (www.predictprotein.org). Interestingly, it is the very sequence that was interrupted by a five amino acid insertion in CHIKV-NCT (insertion occurred after Pro-residue), clearly indicating the importance of this region for the phenotype of the CHIKV replicon. However, it is not clear to what degree the nuclear transport (or the lack of it) contributes to the non-cytotoxic phenotype of CHIKV-NCT replicons. We have demonstrated that in cells transfected with the wild-type replicon (CHIKV-LR), a significant amount of nsP2 was found in the nuclei. In contrast, a lower degree of nuclear localization of nsP2 was generally observed in cells transfected with CHIKV-NCT replicon (Fig. 2B). The direct comparison of these phenotypes was, however, not possible due to the different replication kinetics of CHIKV-RL and CHIKV-NCT replicons (Fig. 2A) and the cytotoxic properties of the former. Thus, the significance of this phenomenon represents a topic of independent study beyond the scope of this report.

The principal difference between the replicon and the infectious virus screening assays used as primary screens is that in the case of an infectious virus assay, chemical agents are allowed to interfere with a system in which the virus is establishing its replicative machinery after entering the host cell. However, in the replicon cell-line based assay, the chemical agent is expected to suppress the activity of already established replication complexes. Considering the rapid onset of alphavirus infection, the need to suppress established replication complexes may resemble more closely the clinical situation, unless the medication is consumed as a prophylactic agent. However, it has been demonstrated that the non-cytopathic replicons of SFV and SINV differ from their wild-type counterparts in that the replication complexes formed by non-cytopathic replicons are unstable and are thus degraded and rebuilt over time [37]. The recycling of the replication complexes also leads to the presence of continuous negative strand RNA synthesis in non-cytopathic replicons, which in the case of wild-type virus is present only early in the infection before the stable replication complexes have been established. In bioactivity screening, the continuous negative strand synthesis may allow the identification of chemical inhibitors also targeting this step in virus replication. Indeed, four of five inhibitors of replication discovered in this study were more potent against BHK-CHIKV-NCT cells than against CHIKV-*Rluc*. However, as the same tendency was also observed for other compounds, including entry inhibitors, it is more likely that this trend was due to the lower sensitivity of the CHIKV-*Rluc* based assay than systems used for primary screens.

Another major difference between the two assays was that the replicon system identifies only inhibitors targeting the replication phase, whereas entry and maturation inhibitors can also be identified in the SFV-*Rluc* infectious virus screen, the time course of which encompasses 2–3 SFV replicative cycles in BHK cells. This feature was also demonstrated by chloroquine used as a reference compound in the study. This antimalarial agent inhibits SFV in the infectious virus assay but has no effect on CHIKV replicon, which is consistent with its well-documented mode of anti-alphaviral action [26], [27]. Furthermore, the SFV-*Rluc* screen identified several hits that did not suppress the CHIKV replicon but were capable of inhibiting CHIKV-*Rluc* infection.

In the current study, new chemical agents with anti-alphaviral properties were identified among both clinically approved drugs and purified natural compounds. Many of the described inhibitors showed similar or superior potency when compared to previously published alphavirus inhibitors. For instance, ribavirin and mycophenolic acid had IC_50_ values of roughly 100 µM in the screening assay, whereas several hit compounds found had IC_50_ values between 10 and 20 µM. In the SFV yield assay, positive controls reduced the virus titers by 1–2 orders of magnitude, while the best hits of this study (apigenin, naringenin, nadoxolol and opipramol) gave results in the same range. With the standard compound 6-azauridine, we were also able to confirm the previously reported differences in sensitivity between alphaviral species towards this compound [16]. Although 6-azauridine suppressed CHIKV replicon with IC_50_ values of 2.4 µM and 3.1 µM (*EGFP* and *Rluc*, respectively) and inhibited CHIKV-*Rluc* (IC_50_ value 29.7 µM), it was able to inhibit SFV-*Rluc* by only 40% at the highest concentration used (200 µM); similar results were obtained in the CPE assay with both SFV and SINV.

Natural compounds with a 5,7-dihydroxyflavone structure (apigenin, chrysin, naringenin and silybin) inhibited CHIKV replicon with IC_50_ values ranging from 22.5 µM to 71.1 µM in a replicon cell line based assay and from 70.5 µM to 126.6 µM in an infectious CHIKV-*Rluc* based assay. Related flavonoids have been reported to inhibit rhinovirus and picornavirus replication, and flavonoids have also been widely studied against HIV [42], [43]. However, to our knowledge this is the first time that their activity has been demonstrated against CHIKV or other alphaviruses. Furthermore, although reports on inhibition of rhinoviruses, picornaviruses and HIV suggest that flavonoids exert their antiviral effects through entry inhibition, the four flavonoids identified here suppressed CHIKV replicon levels with no effect on SFV entry. These results indicate that their target site against these viruses is replication rather than entry.

When the chemical structures of the identified inhibitors were examined, 10*H*-phenothiazine core was identified in six out of twelve pharmaceutical compound hits (Fig. S1). IC_50_ values ranging from 11.3 µM to 25.1 µM were determined for these compounds against SFV-*Rluc*. When testing the compounds in the SFVts9 entry assay, they were demonstrated to effectively inhibit SFV entry into BHK cells, which was also consistent with the fact that they did not have any effect on CHIKV replicon expression levels but did inhibit the infection of CHIKV-*Rluc*. Chlorpromazine, one of the six 10*H*-phenothiazines assayed, was recently reported to also inhibit hepatitis C virus entry, and this compound has been previously reported to inhibit clathrin-mediated endocytosis by preventing the formation of clathrin-coated pits at the plasma membrane [44]–[46]. Clathrin-mediated endocytosis is the main route by which alphaviruses and several other enveloped viruses enter their host cells. The observed inhibition of SFV entry is likely the consequence of misassembly of clathrin lattices in the presence of chlorpromazine. Besides chlorpromazine, we identified five other clinically approved drugs sharing the same 10*H*-phenothiazine backbone that also inhibited SFV entry. This finding suggests that interference with clathrin-mediated endocytosis is a property common for these closely related structures and that clathrin-mediated endocytosis may be a viable target for novel entry inhibitors against alphaviruses and other virus species relying on this mechanism. Furthermore, many clinically approved drugs carrying this structure are indicated for psychiatric or neurological disorders, showing that this chemical scaffold may be a viable starting point for identification of therapeutic agents capable of crossing the blood-brain barrier. The relevance of this aspect in the treatment of CHIKV is currently under debate. Although recent studies have suggested that CHIKV is able to infect neuronal cells and the 2005–2007 outbreaks were accompanied with high numbers of pediatric CHIKV patients with neurological symptoms, the animal models for CHIKV have shown no positive immunostaining in the brains of the infected animals [11], [13], [47], [48].

In conclusion, the current study presents the selection of a stable BHK-based cell line harboring CHIKV non-cytotoxic replicon and its successful use for inhibitor screening. Additionally, evidence on the validity of SFV as a surrogate virus species for screening of possible CHIKV inhibitors was demonstrated by consistent results with the two screening campaigns presented and by verification of selected hits using infectious CHIKV-*Rluc*. A novel virus entry assay is presented using a ts-mutant of SFV at elevated temperature. Inhibitors of alphavirus replication showing two new lead structures, 10*H*-phenothiazines and 5,7-dihydroxyflavones, were identified, the former inhibiting virus entry and the latter preventing intracellular replication.

## Materials and Methods

### Cells

Baby hamster kidney BHK21 cell line was purchased from the American Type Culture Collection (ATCC CCL-10). The cells were grown in Dulbecco's Modified Eagle's Medium (DMEM) supplemented with 8% fetal calf serum (FCS), 2% tryptose-broth phosphate, 2 mM L-glutamine, 100 IU/ml penicillin and 100 µg/ml streptomycin. The CHIKV replicon-containing BHK-CHIKV-NCT cell line was maintained in the same medium supplemented with 5 µg/ml puromycin.

### CHIKV replicon cell line

A plasmid containing the cDNA of a CHIKV-La Réunion (LR) replicon that was used as starting material for the construction of stable BHK cell lines harboring the non-cytotoxic (NCT) CHIKV replicon was kindly provided by Dr. Stephen Higgs (University of Texas Medical Branch, Galveston, TX, USA). This replicon is based on the LR2006 OPY1 strain of CHIKV, which was originally isolated from the serum of a febrile French patient returning from La Réunion Island [19]. A cassette encoding Pac fused to *EGFP* via the 2A autoprotease element of FMDV was inserted under the control of the sg-promoter of the CHIKV replicon. Second, a mutation previously described to reduce the cytotoxicity of the replicons of the related SFV [22] was introduced into the nsP2 coding region (in the case of CHIKV the change was P718 to G). The resulting mutant was designated as CHIKV-PG. The mutation identified by sequencing of viral RNA obtained from a cell clone stably harboring the CHIKV replicon was introduced into CHIKV-PG by site-directed mutagenesis (see Fig. 1A in the Results section). In addition, the coding sequence of *Rluc* was inserted into the replicon vector after the codon for amino acid 1823 of P1234 reading frame (after codon 490 of nsP3). The resulting construct was designated CHIKV-NCT and used for *in vitro* transcription and subsequent transfection of BHK cells.

Confocal immunofluorescence microscopy was performed using a Leica TCS SP5 confocal microscope with a HCX APO 63× glycerol objective, as described in [24]. A mouse monoclonal antibody against dsRNA (J2) was purchased from Scicons (Hungary). For the analysis of subcellular localization of wild-type and mutant forms of nsP2, the BHK cells were transfected with *in vitro* synthesized transcripts of CHIKV-LR, CHIKV-PG and CHIKV-NCT replicons using the Lipofectamine 2000 reagent (Invitrogen, USA), fixed at 8 h or at 16 h post-transfection and stained with 4′,6-diamidino-2-phenylindole (DAPI) and rabbit polyclonal antibody against nsP2 of CHIKV (prepared in house).

For Northern blot analysis, 1×10^6^ BHK cells were transfected with 50 µg of *in vitro* transcribed RNA of CHIKV-LR, CHIKV-PG or CHIKV-NCT replicons or that of their variants containing the *Rluc* marker in the nsP3 region. At 16 h post-transfection, the total RNA was isolated from the cells using Trizol reagent (Invitrogen, USA) and analyzed as previously described [22] using a P^32^-labelled RNA probe complementary to the 3′-UTR region of CHIKV.

### Viruses

Wild-type SFV and SINV stocks were derived from the infectious clones pSFV4 and pTOTO1101 [49], [50] as described in [30]. The working stocks were titrated, yielding titers of 4.5×10^9^ plaque forming units (PFU)/ml and 1.2×10^9^ PFU/ml for SFV and SINV, respectively. SFV-*Rluc*, an SFV strain containing the *Rluc* insertion, was produced from the infectious clone SFV-*RlucH2* [30]. This stock was propagated similarly, yielding a working stock with 1.5×10^9^ PFU/ml. SFVts9-*Rluc* virus, a ts-mutant with a point mutation in nsP2 [31], [32], was modified to include *Rluc* in a manner identical to SFV-*Rluc*. The virus stock was produced via electroporation with the corresponding *in vitro* transcribed RNAs into BHK cells. Plated cells were incubated at 28°C for 48 h. The collected stock of SFVts9-*Rluc* was characterized for the ts-phenotype.

The full-length infectious cDNA clone of CHIKV-LR2006 OPY1 was constructed from synthetic cDNA fragments (Geneart AG, Germany) and fragments originating from cDNA clone of a Mauritius isolate of CHIKV, kindly provided by Dr. Beate Kümmerer (University of Bonn, Germany). The *Rluc* marker was inserted into the region encoding nsP3 similar to the method used for *Rluc* insertion into the CHIKV-NCT replicon. The resulting clone was designated pCHIKV-*Rluc*. The virus was rescued from *in vitro* produced transcripts in BHK-21 cells and checked for genetic stability (indicated by the presence of the *Rluc* marker). The working stock of CHIKV-*Rluc* was plaque-titrated in BHK cells, yielding titers of 6.8×10^7^ PFU/ml. MEM supplemented with 0.2% bovine serum albumin (BSA) and 20 mM HEPES (pH 7.2) was used as the medium for all infections.

### CHIKV replicon screening assay

Before using the BHK-CHIKV-NCT cells for screening the assay set-up was optimized and validated by testing different conditions, such as seeding densities, incubation times and serum concentrations (data not shown). In the optimized format with EGFP detection, inter-plate and inter-day variations in the normalized mid-signals were 4.7% and 7.4%, respectively. For the screening, the cells were seeded onto opaque-white 96-well plates with clear bottom at 3×10^4^ cells/well. The cells were exposed to the test compounds after overnight incubation at 37°C. Compound stocks were diluted in DMEM supplemented with 20 mM HEPES (pH 7.2), 5% FCS, 2 mM glutamine, 100 IU/ml penicillin and 100 µg/ml streptomycin. In a standard assay, 48 h exposure was used prior to replicon expression readout. *Rluc* expression was determined with a *Renilla* luciferase assay kit (Promega, USA) according to the manufacturer's instructions (luminescence was recorded using 1 -s measurements and automatic dynamic range setting). Before *EGFP* detection, the cell cultures were washed with PBS and left with 100 µl of PBS for the measurement. The *EGFP* signal was read at 478/508 nm (excitation/emission) using a 5 nm band width. Fluorescence and luminescence measurements were performed using a Varioskan Flash plate reader (Thermo Fischer Scientific, Finland).

### SFV-*Rluc* and CHIKV-*Rluc* assays

A recently reported anti-alphaviral screening assay [29] was used to determine inhibition of virus infection in cell cultures. Briefly, confluent BHK cell cultures in 96-well plates were infected with SFV-*Rluc* (MOI 0.001), and each library compound was added into the wells simultaneously with the virus inoculum. At 14 h post-infection, the cultures were washed with phosphate-buffered saline (PBS) and 20 µl of lysis reagent (Promega, USA) was pipetted into the wells. The *Rluc* activity resulting from the translation of SFV-*Rluc* genomic RNA was determined from the lysates using a *Renilla* luciferase assay kit (Promega, USA) with a Varioskan Flash plate reader as described above. For dose-response experiments, a dilution series with concentrations of 0.01 µM, 0.1 µM, 1 µM, 5 µM, 10 µM, 25 µM, 50 µM, 100 µM and 200 µM was used for each of the screening hits. Similar conditions were used for confirmation of the hits in CHIKV-*Rluc* assay except that *Rluc* activity was measured at 16 h post-infection using a Glomax 96 microplate Luminometer (Promega, USA).

### CPE reduction assay

CPE reduction was assayed using confluent BHK cell cultures in 96-well plates infected with either wild type SFV or SINV (MOI 0.01) in the presence of primary screen hits at various concentrations. After optimizing the infection times (22 h for SFV and 24 h for SINV), the cultures were washed twice with Hank's balanced salt solution and 10 µl of WST-1 Cell proliferation assay reagent (Roche Diagnostics, Germany) was added. After 1 h incubation, the absorbance at 440 nm was measured to detect the presence of the reduced formazan product using a Varioskan Flash plate reader.

### Viral yield analysis using a plaque assay

BHK cells cultured on 35-mm dishes were infected with wild-type SFV (MOI 0.01) in the presence of 50 µM hit compounds and viruses were collected from the culture medium 16 h post-infection. The viral yields from the collected medium samples were titrated by infecting BHK cells on 6-well plates with serial dilutions of each sample. After 1 h virus adsorption, the cultures were washed and incubated for 48 h in MEM supplemented with 4% FCS, 2 mM glutamine, 20 mM HEPES (pH 7.2), 100 IU/ml penicillin and 100 µg/ml streptomycin and 0.45% carboxymethyl cellulose. Afterwards, the cultures were washed with MEM + 0.2% BSA and stained with crystal violet for quantification of plaques produced by each dilution.

### Entry inhibition assay

Confluent BHK cell cultures in 96-well plates were infected with SFVts9-*Rluc* after equilibrating the cell cultures at 39°C. The infected cultures were kept at 39°C until they were washed with PBS, lysed and *Rluc* activities measured as described above.

### Cell viability assay

BHK and BHK-CHIKV-NCT cells were seeded onto 96-well plates at densities of 3×10^4^ cells/well, incubated overnight and treated with the hit compounds at various concentrations. After exposure for 48 h, cellular levels of ATP were determined as a measure of cell viability after compound exposure [51]. Briefly, plates were equilibrated to room temperature and 100 µl of CellTiter-GLO® luminescent cell proliferation assay reagent (Promega, USA) was added. After 10 -min of shaking, the luminometric readout was measured using a Varioskan Flash plate reader as described above.

### Compound libraries

Two compound libraries were included in this study: a collection of 123 natural compounds, and a library of 233 pharmaceutical compounds. The natural compound library consisted of commercially available pure natural products and their synthetic derivatives, mainly flavonoids, coumarins and other phenolic compounds. The collection of pharmaceutical compounds contained clinically approved drugs in different therapy areas as well as some metabolites of the drugs and other pharmaceutical reagents. All compounds were obtained from commercial sources (Table S1), dissolved in dimethyl sulfoxide (DMSO) and stored as 20 mM stock solutions. The complete list of compounds is presented in Table S1 along with the primary screen data.

### Reference compounds

Five previously published alphavirus inhibitors were used as positive controls in this study. Ribavirin, mycophenolic acid, chloroquine and 6-azauridine [16], [26], [42], [52] were purchased from Sigma-Aldrich (USA), and 3′-amino-3′-deoxyadenosine [30] was a gift from Prof. Seppo Lapinjoki (University of Kuopio, Finland). These compounds were also dissolved in DMSO and stored as 20 mM stocks.

### Data analysis

All data from the antiviral and cell viability assays were normalized using untreated infections and noninfected cell cultures (in antiviral assays) or nontreated and reagent background samples (in cell viability assays), which were set as 100% and 0% values, respectively. Antiviral and cell viability IC_50_ values were determined by fitting the results from dose-response studies into sigmoidal dose-response curves with GraphPad Prism 5.0 software.

## Supporting Information

Figure S1 Chemical structures of 10*H*-phenothiazines identified as SFV entry inhibitors.
(DOCX)

Table S1 Primary screening of chemical libraries. Compounds were tested at 50 µM concentration. See Materials and Methods of the article for experimental details.
(XLS)
