# Supplementary material for: Inhibitors of Alphavirus Entry and Replication Identified with a Stable Chikungunya Replicon Cell Line and Virus-Based Assays
Source: PLoS One. 2011 Dec 19;6(12):e28923. doi: 10.1371/journal.pone.0028923 (PMC3242765; doi:10.1371/journal.pone.0028923)
Supplement: Figure S1 — Chemical structures of 10H-phenothiazines identified as SFV entry inhibitors. (DOCX) [file pone.0028923.s001.docx]

**Figure S1.** Chemical structures of 10*H*-phenothiazines identified as SFV entry inhibitors.

| **Compound** | **R_1_** | | **R_2_** |  |  |
| --- | --- | --- | --- | --- | --- |
| Chlorpromazine | Cl- | | CH_2_CH_2_CH_2_N(CH_3_)_2_ |  |  |
| Ethopropazine | H | | CH_2_ C(HCH_3_)N(CH_2_CH_3_) _2_ |  |  |
| Methdilazine | H | |  |  |  |
| Perphenazine | Cl |  | | | |
| Thiethylperazine | SCH_2_CH_3_ |  | | |  |
| Thioridazine | SCH_3_ |  | | |  |
